# Going beyond established model systems of Alzheimer’s disease: companion animals provide novel insights into the neurobiology of aging

**DOI:** 10.1038/s42003-023-05034-3

**Published:** 2023-06-21

**Authors:** Alexandra A. de Sousa, Brier A. Rigby Dames, Emily C. Graff, Rania Mohamedelhassan, Tatianna Vassilopoulos, Christine J. Charvet

**Affiliations:** 1 Centre for Health and Cognition, Bath Spa University, Bath, UK; 2 Department of Psychology, University of Bath, Bath, UK; 3 Department of Computer Science, University of Bath, Bath, UK; 4 Department of Biology and Biochemistry, Milner Centre for Evolution, University of Bath, Bath, UK; 5 Department of Pathobiology, College of Veterinary Medicine, Auburn University, Auburn, AL USA; 6 Department of Anatomy, Physiology and Pharmacology, College of Veterinary Medicine, Auburn University, Auburn, AL USA

**Keywords:** Cognitive ageing, Neural ageing

## Abstract

Alzheimer’s disease (AD) is characterized by brain plaques, tangles, and cognitive impairment. AD is one of the most common age-related dementias in humans. Progress in characterizing AD and other age-related disorders is hindered by a perceived dearth of animal models that naturally reproduce diseases observed in humans. Mice and nonhuman primates are model systems used to understand human diseases. Still, these model systems lack many of the biological characteristics of Alzheimer-like diseases (e.g., plaques, tangles) as they grow older. In contrast, companion animal models (cats and dogs) age in ways that resemble humans. Both companion animal models and humans show evidence of brain atrophy, plaques, and tangles, as well as cognitive decline with age. We embrace a One Health perspective, which recognizes that the health of humans is connected to those of animals, and we illustrate how such a perspective can work synergistically to enhance human and animal health. A comparative biology perspective is ideally suited to integrate insights across veterinary and human medical disciplines and solve long-standing problems in aging.

## Abstract (web summary)

In this Perspective, the authors illustrate how the use of companion animal models will provide insight into of the neurobiology of aging.

## Introduction

**Fig. 1:**
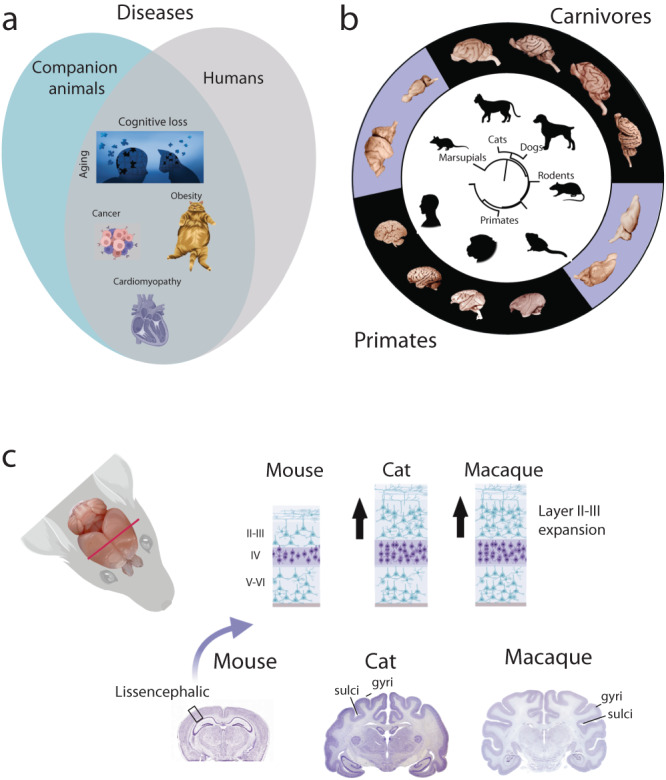
Companion animals share many commonalities with humans. **a** Humans and companion animals suffer from many overlapping diseases. Those include cognitive aging, obesity, cancer, and hypertrophic cardiomyopathy. Age is one of many factors in the development of these diseases. A One Health perspective advocates that individuals from veterinary and human medicine working collaboratively across fields will synergistically enhance the health of humans and animals. **b** Phylogeny of model systems and associated brains from each Order. Rodents, marsupials, and NHPs (e.g., macaques, marmosets) are used as model systems to understand human aging. Although companion animals (i.e., cats, dogs) are more distantly related to primates than rodents are to primates, companion animals share many similarities with humans. **c** Carnivores and primates both possess gyrencephalic brains. A close-up of the cortical gray matter highlights similarities in cytoarchitecture (e.g., an expansion in upper layers) in cats and macaques relative to rodents^36,39^.

One Health is the concept that cross-disciplinary approaches that bring humans, animals, and their shared environments together can synergistically resolve problems afflicting humans and animals^1^. One Health recognizes that the health of humans is connected to those of animals and the environment. One Health is frequently discussed in the context of infectious diseases. For example, environmental degradation and ecological disruption can promote zoonotic diseases (i.e., diseases transmissible across species), which negatively impact human health. A One Health approach has much broader implications for human health than simply reducing zoonotic transmission^2–4^. A One Health perspective can positively impact our understanding of non-transmissible diseases shared by humans and other species (Fig. 1a).

The fields of evolutionary and comparative biology are ideally suited to bridge veterinary and human medicine disciplines to improve our understanding of diseases affecting humans and animals^3^. Companion animals, which are domesticated dogs and cats maintained as household pets (Table 1 and Supplementary Data 1), suffer from many diseases also observed in humans. Like humans, companion animals suffer from cancer, aging, obesity, heart disease, diabetes, and kidney disease^5^. Veterinary and human medicine are focused on identifying and treating similar diseases, but these two fields rarely interact. Nevertheless, there are ground-breaking examples where treatments developed for companion animals have yielded concrete benefits to human patients^6–11^. Obesity, brain aging, retinal aging, and cancer are a few examples of the many areas in biology that stand to gain from increased exchanges between the fields of human and veterinary medicine (Fig. 1a). For example, cats have proven to be excellent models for ophthalmological conditions because of similarities with humans, including high genomic diversity, with the Abyssinian cat breed proving a ‘naturally’ occurring model of retinal degeneration^12^. We illustrate how a One Health approach could expand our toolkit to solve enduring challenges in the neurobiology of aging.

**Table 1:** Keywords used for purposes of this article.

Keywords:
Companion animal models: are domesticated cats and dogs, which are species typically maintained as household pets. Some studies referenced in this review are from animals housed in laboratory colonies.
Nonhuman primate (NHP) models: are defined as any primate species used as primate model systems, such as macaques, marmosets, and lemurs.
Traditional mice and rat models: are laboratory domesticated rats and mice (genera: *Rattus, Mus*), which are most frequently used for study in biomedical sciences (see Fig. 3).
Carnivore: are any wild or domesticated animal classified as belonging to the order Carnivora (e.g., cats, dogs, ferrets, polar bears, cheetahs, and red pandas).
Domesticated animals: is defined as any animal that is maintained as a companion, pet, or is raised on a farm.

The definitions of these terms vary in official and common usage (see Supplementary Data 1).

Alzheimer’s disease (AD) is a disease of aging and is characterized by brain plaques and tangles, brain atrophy, and cognitive dysfunction^13–15^. AD was traditionally thought to be unique to humans though this interpretation is based on observations made from a handful of model systems^16,17^. This limited range of species spans rodents (i.e., rats, mice; Table 1), which are widely used in biomedical sciences (Fig. 1b; See Supplementary Methods). We also consider nonhuman primates (i.e., NHPs^18^) used in research (e.g., marmosets, mouse lemurs, African green monkeys, macaques, and chimpanzees) and companion animal models (Fig. 1b). NHPs have commonly been considered the model organisms that most closely resemble human behavior and disease profiles due to their psychosocial and genetic similarities^19^. Rodent and NHP model systems recapitulate limited aspects of AD, but companion model systems (i.e., carnivores used as companion animals) are emerging as valuable models to understand human aging in health and disease.

Companion animal model systems are here defined as domesticated cats (*Felis catus*) and dogs (*Canis lupus familiaris*), which are species that are maintained as common household pets though they are sometimes used in the laboratory (Table 1 and Supplementary Data 1)^20^. Companion animals are rarely considered model systems to probe human biology predominantly *because they are our companions*, and there are additional regulations for using cats and dogs (and also primates) as model systems in research compared to rodent models. For example, the U.K. Government’s Home Office classifies them as “Specially protected species” which can only be used for research cases in the absence of any other suitable model system^21^. The U.S. Government’s Animal Welfare Act was put in place largely to protect cats and dogs to which it applies (and also primates), but this does not apply to common laboratory strains of rodents^22^ (Supplementary Data 1). Cats and dogs encapsulate a diverse group of animal breeds; they are thought to have been domesticated over the past 4–30,000 years^23,24^, with a conservative review suggesting dogs were domesticated 15,000 years ago^25^ in Europe or Asia and cats were domesticated 4000 years ago^25^ in the eastern Mediterranean Basin of Northern Africa and the Near East.

A similarity between domesticated animals and humans is that they occupy the same (built) environments that humans do. The term domestic comes from domus, which is Latin for house. Yet until recently domesticated animals have lived around, but outside of, human homes. One study found that although dogs lived among humans in 53 of 60 cultures studied, they were allowed inside human houses only in seven of them^26^. Practices have been changing in recent decades, however. In the 1960s a quarter of dogs in the USA (owned and unowned) roamed the streets, but this was reduced when animal sheltering started in the 1970s^27^. While cats and dogs have been domesticated for millennia, they previously endured much work and/or hostility from humans, but they are now typically house-dwelling and treated as members of the family^26^. This factor might link companion animals to humans more than other domesticated species.

On the other hand, Darwin^28^ suggested that domesticated species (including both companion animals and farm animals) share a suite of anatomical, physiological, and behavioral traits, to the exclusion of their wild ancestors. This so-called *domestication syndrome* may be caused by selection for reduced aggression in mammals, which may result in changes in the expression of genes that direct neural crest cell development^29^. It has further been suggested that the domestication of some species, including dogs and cats, cannot be attributed entirely to human actions. Instead, it has been suggested that they were *self-domesticated* and had autonomously come to occupy domesticated niches^30^. Dogs may have participated in their own domestication from wolves by benefiting from being near human habitation sites^31^. Cats may have been active in their own domestication by lurking near human habitations, while maintaining contact with the wild population and having control of their own resource supply, a process dubbed *semi-domestication*^32^. It has even been suggested that humans may have self-selected for tameness and that the same genetic factors contributed to reduced craniofacial features compared to our closest relatives the Neanderthals^30^. It remains uncertain to what extent the domestication *process* in cats and dogs was different than in livestock species and akin to something that took place in recent human evolution. However, here we propose that the similar environments of humans and companion animals make them especially well-suited to study human biology.

Comparative biology is an important area of study because it considers the natural variation that has arisen through evolutionary pressures. We can use information from comparative neuroscience research to identify the model systems best suited to aging^33–35^. Companion animals share many similarities in brain organization (Fig. 1c) and possess age-related changes in biology and behavior that resemble humans^36–40^. Companion animals also share very similar environments (e.g., shared sleeping quarters, diets, experiences, toxins, stressors) with their owners. The close relationship of companion animals to humans combined with their relatively shortened life makes them well-suited to monitor how the environment impacts health and rates of aging^41–43^. Companion animals are also advantageous to study because some of them are genetically homogenous populations in purebreds. Others are genetically heterogeneous outbred populations in mixed breeds^44^. The pattern of variation across these different populations means that we can investigate the interaction of genotypes and toxins, stressors, or diseases on rates of aging. Similarities in these traits point to companion animal models as valuable models to study aging.

We discuss the structural, molecular, and behavioral changes in companion animal models during aging. More specifically, we discuss brain atrophy, plaques, tangles, and memory impairments across species. We evaluate which model systems demonstrate age-related changes in behavior and biology^45–48^. Our comparative analyses showcase that companion animal models spontaneously accrue many of the hallmarks of human aging. These observations prompt the need for increased communication across human and veterinary medicine. These synergies may resolve challenges in the study of aging and other long-standing biomedical issues.

## What is Alzheimer’s disease?

Alzheimer’s disease (AD) is a progressive, fatal brain and behavioral disease that impairs memory and cognitive skills. AD also includes non-cognitive psychiatric symptoms such as irritability, and loss of normal sleep patterns^49–51^. Historically, AD has been considered unique to humans because it has been elusive in a handful of studied model systems. AD accounts for 50–80% of dementia cases and, accordingly, is one of the most common types of dementia^52^. Some records indicate ancient Egyptians may have documented AD^53^. Still, it was not until 1906 that Alois Alzheimer discerned neuritic senile plaques and neurofibrillary tangles in the autopsied brain of 51-year-old Auguste Deter, who suffered from amnesia and dementia^54^. In humans, AD is classified as early-onset if the disease emerges earlier than 65 and late-onset if it arises at 65 and over^55^, and the latter makes up around 95% of cases^56^. Clinically, individuals with AD experience irreversible and progressive behavioral disabilities, including cognitive dysfunction, memory loss, and brain pathologies^57^. We describe each of these dysfunctions in AD and consider how age-related changes map onto rodent, primate, and companion model systems.

## Cognitive loss in AD

The most salient aspect of the disease is cognitive loss. The disease usually begins subtly and progresses sequentially (stages 1–3). Initial symptoms include forgetting names, locations, events, and misplacing objects (i.e., mild stage 1^54^). Individuals then become disoriented and eventually fail to recognize family members (i.e., moderate stage 2^54,58^). Finally, AD patients have difficulty writing, reading, speaking, and concentrating. They become restless, depressed, withdrawn, and lose basic functioning abilities in their final years (severe stage 3^54,58^). Cognitive aging has also been reported in diverse nonhuman animal species and includes memory loss, decreased attention span, reduced performance on spatial tasks, anxiety, locomotor disabilities, as well as changes in vocalizing^46,48,59–71^.

## Neuropathologies and brain atrophy in AD

**Fig. 2:**
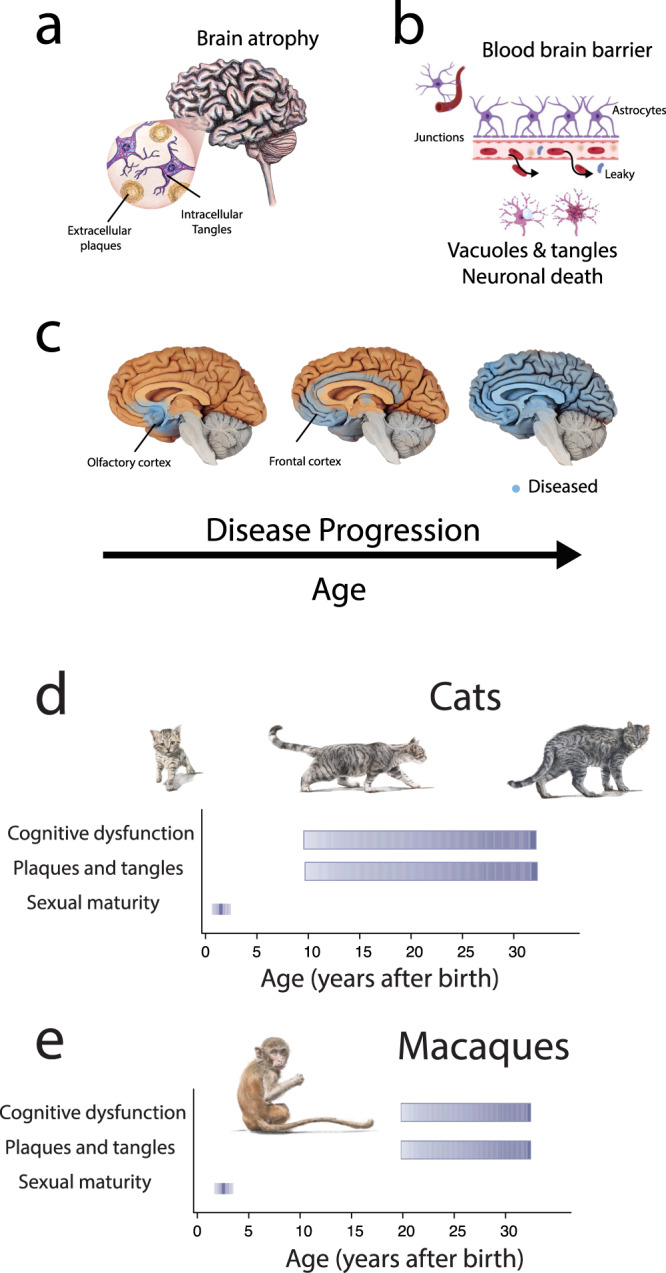
Alzheimer’s disease is associated with a specific range of pathologies, which emerge at different ages in different species. **a** Major hallmarks of AD include brain atrophy, plaques, and tangles (NFTs). **b** The disease is characterized by a leaky blood-brain barrier. Normally, astrocytes form tight junctions, so much so that these barriers formed by astrocytes prevent the entry of materials into the brain. In AD, the blood-brain barrier is compromised, and the disease is also characterized by vacuoles and neuronal death. **c** There is a stereotypic sequence in the accumulation of brain plaques and tangles starting in the olfactory cortex and hippocampus, which then spread through most of the brain. The early accumulation of plaques and tangles in the olfactory cortex is concomitant with the loss of olfactory abilities. We compare the timeline of brain plaques and tangles in cats (**d**) and macaques (**e**). Cats may accumulate plaques and tangles for a relatively long percentage of their life. Many reports indicate that cats can develop plaques and tangles and cognitive dysfunction before the age of 10, but similar deficits are more likely expected to occur in macaques in their 20s^38,174^. Cats recapitulate the hallmarks of aging relatively early and so may be an ideal model system of aging. Dashed lines represent a probability of occurrence and are based on qualitative reports from the literature (see Supplementary Data 2)^125^.

Specific neuropathologies characterize AD though it is still unknown whether these pathologies are side effects of the disease process or their cause^72,73^. The accumulation and deposition of amyloid beta (Aβ) plaques and neurofibrillary tangles (NFTs), which are formed by hyper-phosphorylation of tau (pTau) proteins (Fig. 2^73^), are hallmarks of the disease^74^. The Aβ peptides are dense deposits of 38, 40, or 42 long amino acid peptides called plaques^73,75^. These extracellular plaques are formed from the breakdown of Aβ precursor protein (*APP*^72,73,76,77^). NFTs are another hallmark of AD and consist of abnormal buildups of twisted strands of intracellular tau (tubulin-associated unit^73,78,79^). In health, tau binds to tubulin, which stabilize microtubules^73,75^. In AD, tau disaggregates from microtubules and bonds to other taus, forming C-shaped paired helical filaments that form relatively long intracellular tangles^73,78,79^, which impact neuronal communication^73,78–80^. These plaques and tangles are first observed in the olfactory and hippocampal regions^72,81–83^ (Fig. 2^81–83^). These neuropathologies subsequently spread through the frontal cortex, the cingulate gyrus, and the rest of the cortex^82^. One of the first symptoms of AD is a loss of smell, which coincides with the presence of neuropathologies (Fig. 2c). Evidence points to the presence of NFTs as a better marker of cognitive decline and disease progression compared to the accumulation of Aβ plaques^84,85^ (Fig. 2c). While extracellular plaques and NFTs characterize the disease, other pathologies are present in AD.

Many aged humans suffer from neuropathologies. Aged humans suffer from brain atrophy, which consists of brain volume decrease, neuronal loss, inflammation, cerebral vascular dysfunction, and neuronal vacuoles^15^. Microglia have also been highlighted as an important cell type for study^13,14,73,76,86,87^. Some plaques, including Aβ40 and Aβ42, are especially toxic because they cause surrounding amyloid groups to form clumps called intermediate oligomers and protofibrils, which are polypeptide masses^72,73,76,88–90^. Plaques and vascular problems may lead to additional diseases, including cerebral amyloid angiopathy (CAA), hemorrhage, atherosclerosis, strokes, and ischemic brain damage^72,73^. Although CAA is highly associated with AD being present in approximately 90% of cases, it also affects between 20–40% of the elderly population with no cognitive impairment^91,92^. Whether these neuropathologies represent a disease that emerges in old age or whether they represent an emergent property of aging remains an open question.

## Potential causes of AD

Many studies have sought to identify genetic and environmental factors, but age remains the top predictor for AD. The likelihood of developing AD doubles every five years after 65^57,93–96^. Nearly half of those over 85 are at risk of AD^54,97^. Besides age, biological sex is a leading predictor for AD in humans, with females at a higher risk of developing AD^98,99^. Past work has not identified strong environmental factors that predict AD^100^. However, it has been suggested that environmental toxins (e.g., formaldehyde from paint, some furniture, and flooring), drug use (e.g., tobacco, alcohol use), diet, a weakened immune system, and hormonal changes impact the likelihood of developing AD^54,58,73,101–104^. AD is also, to some extent, comorbid with hypertension, diabetes, hyperlipidemia (i.e., a disease characterized by an excess of fat in the blood), stroke, sleep apnea, cardiovascular disease, and metabolic syndrome^95^. Interestingly, there is an inverse relationship between educational attainment and AD risk^54,105^. However, this may, in part, be due to cognitive decline measures not being sensitive enough for those who are highly practiced at assessments, potentially leading to a late diagnosis^106,107^. Thus, the threshold set to diagnose the disease could be adjusted depending on the individual’s background^107,108^.

After the age of 80, females are at higher risk of developing AD than males^109,110^, and several factors are likely influencing biological sex differences in AD incidence risk. Females live longer than men, and the relatively extended lifespan in females may elevate the risk of developing AD^111^. Other factors are likely important in dictating sex differences in disease risk. For example, females are at higher risk of developing diseases such as obesity and diabetes that predispose them to AD^112^. Alternatively, or in addition, hormonal changes during menopause may predispose females to contract AD^109,112,113^. Several female-specific biological processes may increase the likelihood that females contract AD.

Family history elevates the risk of AD^54,93,114^. Mutations in key genes predispose individuals to AD. Those include amyloid beta precursor protein (*APP*), presenilin 1 (*PSEN1*), and presenilin 2 (*PSEN2*), which contribute to early-onset types of AD. Other gene variants (e.g., *SORL1*, *TREM2*, *ABCA7*, and apolipoprotein E4: *APOE4*) and long non-coding RNA gene variants contribute to AD^54,57,58,73,93,104,115–118^. One gene called *APOE* comes in several different alleles, including *APOE2*, *APOE3*, and *APOE4*. *APOE3* is neutral and is the most common allele (observed in 75% of the population); *APOE2* reduces the risk of AD, but *APOE4* is associated with an increased risk of AD^119^ and late onset of the disease^16,120^. Female carriers of *APOE4* have a higher risk of developing AD than male carriers^121^. *APOE*4 is the ancestral *APOE* allele and is similar in sequence to the common *APOE* allele in companion animals and non-human primates^17,122,123^. A commonly studied gene called *APP* plays an integral role in forming plaques and tangles because *APP* produces a transmembrane protein, which gives rise to amyloid-β (Aβ) peptides. *APP* mutations lead to abnormal amounts of Aβ^54^ and brain plaque formation^54,86,93,116,124^. Much effort has focused on manipulating *APP* levels in rodent models to characterize how *APP* leads to plaque formation and cognitive deficits.

## Down syndrome informs the etiology of AD

Individuals with Down syndrome are at high risk of developing AD. Down syndrome patients have a third copy of chromosome 21 and a concomitant third copy of the *APP* gene^93^, which produces large amounts of amyloid-β (Aβ) peptides. Individuals with Down syndrome exhibit many of the AD phenotypes present in the general population, but they do so on an accelerated timeline relative to general AD patients (Supplementary Data 2, 3^125^; Supplementary Methods). Variation amongst individuals with Down syndrome informs the role of *APP* in the development of AD.

Individuals with Down syndrome accumulate relatively mature Aβ deposits (Aβ42 > Aβ40), microgliosis, astrocytosis, neuritic dystrophy, and neurofibrillary tangles early on, and they possess brain atrophy, especially in the amygdala, hippocampus, and the white matter^126^. Together, these phenotypes resemble those of AD patients from the general population^126,127^. Unlike general AD patients, however, individuals with Down syndrome and AD demonstrate behavioral and personality changes not clearly observed in other AD populations^126^. What is most striking is the early timeline of AD in Down syndrome (Supplementary Data 2, 3^125^).

Individuals with Down syndrome are at much higher risk of developing AD, and at relatively early ages. The average onset age of AD in Down syndrome is in the mid 50s with a mean age of death set to late 50s^128^. As much as 80% of Down syndrome patients with AD have plaques and tangles in their brain by their 40s and these can appear in the teens^126^ (Supplementary Data 2). The third copy of the APP gene on chromosome 21 is associated with increased and accelerated rates of plaques and tangles.

Some individuals with Down syndrome have a translocation that eliminates the *APP* gene in the replicated portion of the gene, and these individuals are less likely to develop the neuropathologies associated with AD than those who do not have such a translocation. Together, these data strongly indicate that the triplication of the APP gene is responsible for the development of AD in Down syndrome^127,129^, and more generally that APP gene is a factor in the development of AD.

### The established model systems: limitations for the study of AD

**Fig. 3:**
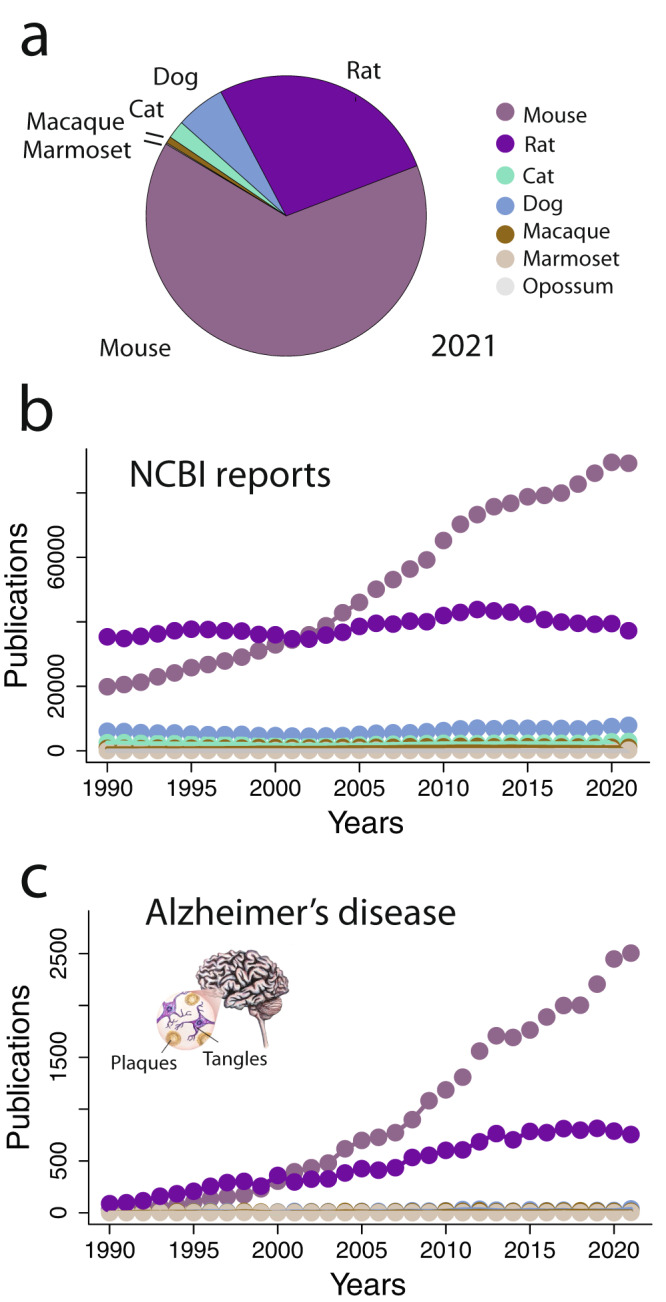
Mice and rats are the most frequently studied model systems. On NCBI, we used species names as keywords and we extracted the number of articles linked to each species. **a** In 2021, rats and mice were utilized substantially more than other model systems. **b** This has been the case for multiple decades. **c** We repeated the search with species name and Alzheimer’s disease as keywords. These data show that mice and rats are the dominant models to understand AD.

Mice have been the model system of choice for studying AD (Fig. 3). In support of this claim, we text-mined NCBI and we extracted the number of articles with keywords containing species names (e.g., *Mus musculus* Fig. 3a, b). We also performed another search, including the species name and the term Alzheimer’s disease, to track the preferred species of study in AD (Fig. 3c). According to these data, mice have been the preferred model system of AD for decades. Indeed, much effort is focused on genetic manipulations of APP in mice to recapitulate aspects of AD. Genetic manipulations are needed to make mice suitable for AD study because mice do not naturally exhibit many aspects of AD. In the following sections, we explore the history of rodents, primates, and companion animals as model systems of human biology. We evaluate how each of these model systems age, whether they age similarly to humans, and we note that companion animal models spontaneously show many similarities to AD.

## A review of mice, rats, and primate model systems

### Traditional mice and rat model systems: strengths and limitations

Rodents have traditionally been used as model systems to investigate the molecular basis of AD. The Norway rat was the first mammal bred as a laboratory model over 170 years ago^130^. Rodent species have been selected as models because they are mammals (rodents shared a common ancestor with humans approximately 87 million years ago^131^) with similar brain features, including a six-layered neocortex (Fig. 1c). Further advantages in research include a short reproductive cycle, thriving in captivity, and being easy to manage^130,132^. In the wild, rats and mice occupy a wide diversity of environments and live among humans. Mice and rats are common household pests. Norway rats, in particular, are associated with the spread of diseases such as the Bubonic plague, so the use of these species has been seen as a redemption for lost human lives^133,134^. While any species could serve as a laboratory model^135^, rats and mice are the only mammals specifically mentioned by the NIH as model organisms^136^. Past research efforts focused on the use of traditional model organisms to facilitate studies on the same species. Our understanding of the role of the hippocampus and entorhinal cortex in spatial memory was founded on research in rodents to identify how specific environmental coordinates correspond to the firing of specific hippocampal place cells^137^ and geometrically positioned entorhinal grid cells^138^. Rodent models have also implicated the role of the hippocampus in social memory^139^.

## Neuropathologies in mice and rat model systems

Despite the widespread usage of rodents, naturally occurring plaques and tangles have generally been challenging to detect in these species so far, suggesting they could be absent^70,140,141^, nor do they exhibit dramatic gross brain loss in old age as humans do^142^. Transgenic mice have been engineered to overexpress beta and tau to recapitulate the biological hallmarks of the disease in humans^143,144^. Some of these models produce human Aβ above physiological levels, leading to a great abundance of senile plaques^143^; however, many fail to develop NFTs and neuronal loss unless mutant tau is also introduced. This limitation is true for the McGill transgenic rat model of AD since NFTs and the component tauopathy are not present^70^. Other models include Aβ-overexpressing Tg344-AD rat models, which do express NFTs formed from endogenous pTau^144^, as well as recurrent HSV-1 infections in mice, which induce both plaques and pTau^145^. Many of the rodent models studied are either adults or at a relatively early stage of life, so age-related changes are sometimes neglected from consideration in these investigations^146^.

## Nonhuman primates as closely related model systems: strengths and limitations

Non-human primates (NHPs) have also been studied in the context of AD^147,148^. The reason for selecting NHPs as model species is because their brain structure and function more closely resemble humans than rodents (Fig. 1c^19,149^). Humans share a common ancestor with all other primates approximately 74 million years ago, with rhesus macaques approximately 29 million years ago, and with our closest relatives – the chimpanzees and bonobos – approximately 6.5 million years ago^131^. Several aspects of the cerebral cortex are shared between humans and rhesus macaques (Fig. 1c; who are both catarrhine primates) to the exclusion of rodent models. Their cerebral cortex is large and folded (gyrified; Fig. 1c). NHPs and humans share similarities, which include their visual systems with an enlarged visual cortex and differentiated frontal and posterior parietal association areas (related to social cognition and dexterity). In addition, NHPs are useful biomedical models of humans due to their behavioral similarities to humans^19^. Among NHPs, rhesus monkeys have been used as models for 130 years, with advantages, including easy maintenance in captivity and rapid development. They thrive in urban areas and have the largest range of any NHP^150^. Their behavior is better studied than any other NHP, particularly through the documentation of a free-ranging island colony that has been studied for 85 years^150^.

There are certainly some disadvantages to using primates as model organisms. No primate species — unlike cats, dogs, and rodents — have been domesticated^151^; however, most primates used in UK research are usually born in captivity^21^. Since these animals have genetic and behavioral adaptations to wild versus domesticated conditions, they have more complex living requirements that create practical (and ethical) issues when keeping them in captivity^151,152^. Despite being the species most closely related to humans, chimpanzees and other great apes (e.g., gorillas, orangutans) are not conventional model organisms and are challenging to maintain in captivity. Nonhuman primates can carry zoonotic diseases (e.g., B virus^153^). Moreover, nonhuman primates share with humans slow development, large bodies, and a dependency on social relationships^154^. Indeed, chimpanzees who experience social and sensory deprivation can develop aimless motions and self-injurious behavior, which can be summarized as stereotypical behaviors^155^. These behaviors are aggravated by physical restraints^156,157^. Self-injurious behavior has also been linked to early separation from mothers in infancy in apes and macaques^157^; this behavior can also develop spontaneously in macaques, whereas this idiopathic development is not typical in rodents^158^. Other monkeys, such as African green monkeys, can display aggressive behaviors, creating challenges when housing them with conspecifics^152^. Furthermore, there are heightened ethical issues with using great apes as models due to their proposed ‘personhood’^159^, which is partly based on their cognitive abilities, such as self-awareness. Unlike dogs and cats, they recognize their reflection in a mirror^160^. Also, great apes have advanced verbal and non-verbal communication^161^ as well as good working and long-term memory^148,154,162,163^. Also, wild populations are not readily available due to their endangered status^164^.

## Neuropathologies in nonhuman primates

Similar to aged humans, NHPs develop Aβ deposits; nevertheless, they lack the profuse manifestation of NFTs typical of AD^16,165^. Among NHPs, cercopithecid species develop senile plaques but rarely develop neurofibrillary tangles^16^. However, this depends on the age of the individual. Specifically, rhesus macaques have an age-related increase in tau phosphorylation (pTau)^166^. Further aggregation of this pTau into NFTs has been observed in particularly aged rhesus macaques, as was the case for a 38-year-old individual who developed NFTs in their entorhinal cortex^147^. Although other cercopithecid species, such as African green monkeys^167^ and cynomolgus monkeys^168^, have occasionally demonstrated NFT-like structures, these have been revealed not to exhibit the biochemical and morphological traits of AD.

Only a few individual NHPs have been observed to have deposits and co-occurring tangles, and this has largely been restricted to chimpanzees. The first case was an obese 41-year-old chimpanzee with high cholesterol who displayed both Aβ plaques and NFTs^169^. Also, only a few have been found to have pathology to the degree observed in humans with AD. For example, a 57-year-old chimpanzee was found to have phase 4 Aβ plaques as well as stage V NFTs^82,170^. That individual was part of a study of twenty chimpanzees aged 39–62 years old, of which only three of the other chimpanzees had NFTs and Aβ deposits co-occurring, but these were within the first phase of the pathology^170^. In contrast, humans clinically diagnosed with AD would typically have phase 3 Aβ deposits and above^171^. These chimpanzees were all younger than the 57-year-old male, suggesting that age, as well as obesity/high cholesterol, are large risk factors in the emergence of the disorder.

One hypothesis is that macaques rarely develop neuropathologies associated with AD because they do not live sufficiently long for these neuropathologies to become manifest. AD pathology, as well as brain atrophy, is difficult to observe in NHPs^16^. Age-related brain atrophy occurs in primates but appears to be relatively modest (e.g., macaques, chimpanzees^142^), though variation in atrophy exists across primate species (e.g., macaques, chimpanzees). Some authors report small (e.g., 5% change) to no significant difference in brain size across the lifespan in chimpanzees^172–174^, but brain atrophy may be pronounced in aged mouse lemurs^67^. Developing new approaches to extend the lifespan of NHPs and other animals falls within the realm of veterinary medicine and may inform what biological programs are products of an extended lifespan and which are unique to humans.

## Companion animal model systems: suitability for AD study

Human brain aging is characterized by brain atrophy, memory, locomotor impairments, and an increased risk of developing certain diseases (e.g., AD). Still, many age-related changes have been observed in rodent and primate models, but some of them have been elusive in rodent and primate models^175^. Rodents and NHPs may not live sufficiently long for some age-related diseases to manifest. Accordingly, rodent and NHP models may not clearly show plaques, tangles, and brain atrophy because their lifespans are shorter than humans. In contrast, companion animals have relatively extended lifespans and exhibit evidence of brain pathologies, atrophy, and cognitive dysfunction. We overview the evidence supporting companion animals as models of aging.

## Evidence of lifespan extension in companion animals

**Fig. 4:**
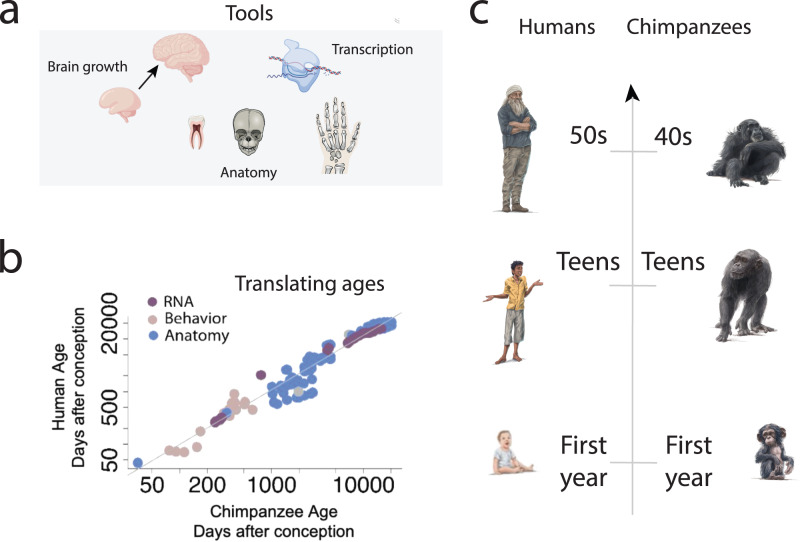
There are evolutionary modifications in lifespans across species. A past study extracted 137 time points from multiple scales of organization, including (**a**) anatomy, transcription, and growth, to generate cross-species age alignments across the lifespan of (**b**) humans and chimpanzees^176,177^. These analyses showcase a surprising degree of similarities between humans and chimpanzees. **c** Shortly after birth, humans are similar in age to chimpanzees in their first year of life. Similarly, a teen human roughly equates to a teen chimpanzee, and a human in their 50s roughly equates to a chimpanzee in their 40s. Most chimpanzees do not live past their 40s such that there is a phase of life in humans with no clear counterpart in chimpanzees. The extended human lifespan relative to other NHPs calls for the need to find species that show a relatively extended lifespan akin to humans to be useful models of geriatry. Figures are modified from^176,177^.

Humans exhibit an extended lifespan contrasted to great apes. Accordingly, biological processes evident in humans may not be observed in great apes simply because the extended lifespan in humans unmasks biological processes not seen in great apes^176^. A recent study aligned ages across the lifespan of humans and chimpanzees^176,177^ (Fig. 4a–c). Age alignments were possible in humans and chimpanzees across prenatal and postnatal stages, but there was no clear age alignment between humans and chimpanzees past 50 years of age; this is because most chimpanzees die in their 40s, which roughly equates to humans in their 50s (Fig. 4c). Accordingly, chimpanzees would not live sufficiently long to recapitulate biological processes that occur in humans in their 50s or later. Broad surveys across mammals are needed to inform which model systems may live sufficiently long lifespans to be useful as model systems of geriatrics.

Many age-related changes occur in elderly humans. Past work identified nine biological processes as hallmarks of aging^178^. Some of these are genetic changes that emerge from genomic instability leading to the increased tendency for mutations and telomere attrition (i.e., the graduate loss of caps at the end of chromosomes). Epigenetic changes, the loss of proteostasis (i.e., the process of regulating proteins), dysregulations in nutrient sensing, mitochondrial dysfunction, cellular senescence, exhaustion in stem cells, and altered intercellular communication are all important factors in aging^178^. More work is needed to compare the biology of humans and other animals at these different scales and to identify how rates of aging in these biological processes have changed across species^179–181^.

**Fig. 5:**
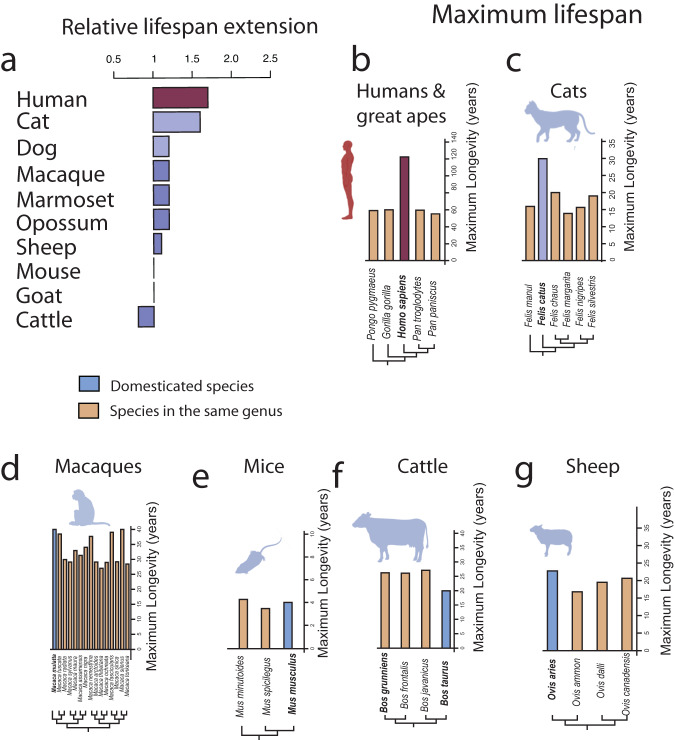
Maximum recorded lifespans across great apes, companion animals, and other domesticated animals. **a** We pooled the average maximum lifespan per genus and divided the lifespan of each species of interest versus the average lifespan per genus. A score above 1 indicates that the domesticated species has an extended lifespan relative to other species in their genus (**a**). Evidently, humans (**b**) possess an extended lifespan relative to others in their genus (**b**). Interestingly, cats (**c**) have also evolved a relatively extended lifespan compared to others in their genus. Macaques (**d**), rodents (e.g., mice) (**e**), and farm animals (**f**, **g**) have not evolved such long lifespans relative to their wild counterparts. These data are from the AnAge database^182^.

We compared the maximum lifespan of different species to assay which, if any, of the model systems possess an extended lifespan relative to their close counterparts (Fig. 5). We used the AnAge database to compare maximum lifespans across hominids, carnivores, traditional model systems, and farm animals (Fig. 5). We compared the maximum lifespan of the domesticated species relative to others within their genus. We pooled the average maximum lifespan per genus, and we divided the domesticated animal’s lifespan relative to the average lifespan within their genus. A score greater than 1 indicates that the model system has an extended lifespan relative to other members of their genus (Fig. 5a). The lifespan is extended in humans relative to great apes, as expected given studies translating ages across these primate species^176^. Of interest, companion animals, especially cats, exhibit a large relative increase in maximum lifespan compared to others in their genus (Fig. 5c). The relative extension in cat lifespan rivals that observed in humans. Commonly studied model systems such as mice, macaques, marmosets, and farm animals such as sheep and cattle exhibit, at best, a modest increase in relative maximum lifespan measured against members within their genera (Fig. 5d–g^182^). Broad comparative analyses such as these highlight that companion animals, especially cats, may be ideal model systems for human aging because of their relatively extended lifespan and the likelihood of an extended period in which to age. In line with this, demographic research has demonstrated that the life expectancy of pet companion animals is increasing^75^, which could explain why a substantial number of cats have been reported to have NFTs compared with NHPs^75^.

## Evidence for brain atrophy in companion animals

**Fig. 6:**
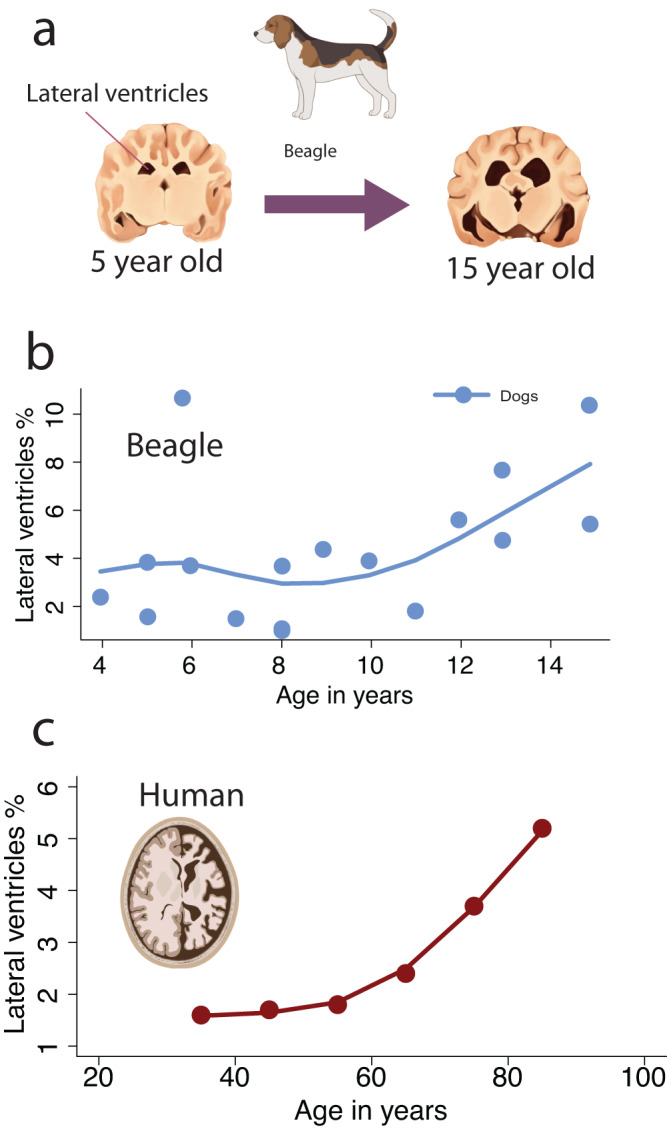
Brain atrophy is evident in aged dogs and in humans. We used the relative volume of lateral ventricles as an index of brain atrophy in dogs (**a**, **b**) and humans (**c**^184^). **b** Plots of the relative lateral ventricles volume versus chronological age show that the lateral ventricles expand with age, but that this largely occurs at 12 years of age and beyond in dogs and at (**c**) 60 years and beyond in humans^183^. The relative lateral ventricle volumes in humans were computed by dividing the lateral ventricle volumes by the cerebral cortex gray, white matter, and lateral ventricle volumes^184^. The dog brains are redrawn from a structural MRI scan of a 2- and 15-year-old beagle available at: https://veteriankey.com/the-effects-of-aging-on-behavior-in-senior-pets/.

Emerging evidence shows that brain atrophy in dogs is as extensive as it is in humans. Here, we considered lateral ventricles (Fig. 6a), which comprise a space that houses cerebrospinal fluid, to measure brain atrophy. The size of the lateral ventricles relative to brain volume expands in beagles with old age, especially beyond 12 years of age^183^ (Fig. 6a, b). In humans, lateral ventricles also enlarge. These age-related changes become particularly evident at around 60 years of age and beyond (Fig. 6c^184^). Although the relative increase in lateral ventricles observed in dogs resembles humans, we have yet to rigorously quantify brain atrophy across humans and companion animals after aligning ages. This work is important because it will enable us to assay the extent to which age-related changes in the brain volume of companion animals map onto humans.

## Cognitive dysfunction syndrome in companion animals

Normal aging is linked to diminished sensory, memory, and cognitive capacities in humans as in companion animals^61,64,65,185–188^. Some aged cats and dogs demonstrate a decline in exploratory behavior and social interaction, and engage in excessive vocalizations^61^. If behavioral and cognitive deficits sufficiently impact daily activities, companion animals can be diagnosed with cognitive dysfunction syndrome (CDS)^64^. Among client-owned pets, the prevalence of CDS may be as high as 22% in geriatric dogs^60,63^ and 50% in geriatric cats^189,190^. These studies further report that CDS is likely underdiagnosed, so the prevalence of age-related cognitive deficits may be higher than that which is frequently reported. These relatively high rates of CDS suggest that it is commonplace for companion animals to suffer from a suite of behavioral and cognitive changes at later stages of life.

Companion animals share many similarities to humans, but cats and dogs also belong to two taxonomic groups with different cognitive and behavioral capabilities^59,69,191–194^. Accordingly, several assessment tools have been designed to monitor age-related impairments in humans and companion animals, but some behavioral and cognitive domains are species-specific. A CDS checklist is used to assess age-related impairments in companion animals^46^. The CDS evaluates whether animals vocalize excessively, house soil, possess abnormal sleep-wake cycles, are anxious, show decreased exploratory behavior, and whether time spent grooming is unusual^46,60,62,64,71,187,195,196^. The rate of occurrence and the number of these behavioral deficits are used to assess the amount of cognitive deficit in companion animals^46,62,71,187,195^. Assessment tools such as the Montreal Cognitive Assessment in humans focus on capturing impairments in emotion, sleep, memory, language, and cognitive capacities^69^. Many of these assessments focus on language and thus assay human-specific cognitive capacities. There are clear behavioral and cognitive impairments at late stages of life in companion animals as in humans, but some of the age-related impairments are species-specific.

## Brain pathology in companion animals

**Fig. 7:**
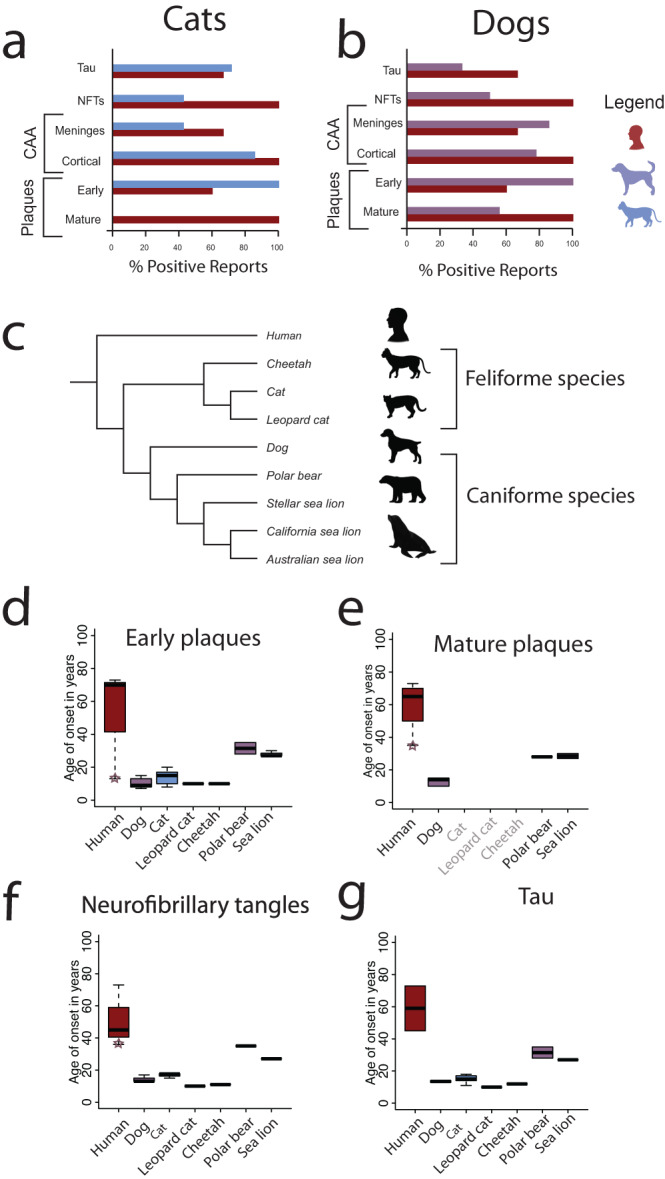
AD associated neuropathologies are found in cats and dogs. We computed the relative number of studies reporting a pathology in humans (**a**, **b**) cats (**a**), and dogs (**b**). These percentages show that many AD-related neuropathologies in humans are also observed in companion animals. Only studies explicitly searching for the presence of **a** neuropathology are considered in these statistics. **a**, **b** Studied pathologies include phosphorylated tau (pTau), NFTs, CAA (i.e., meningeal, and cortical), as well as early and mature plaques. A relatively large percentage of studies report plaques, pTau, and NFTs in cats and dogs. **a** However, no mature plaques are reported in cats. These data are from Supplementary Data 2^125^. Age distribution of neuropathologies onset across humans and carnivore species (**c**–**e**), including companion animals and **e** other carnivore species. We considered the age of onset of early (**d**) and Amyloid-β e as well as tau pathology (**f**) and tau (**g**) from data provided in Supplementary Data 2. We included all listed ages (i.e., ≥ and >), as well as data from individuals with Down syndrome. The age of early and mature plaques, as well as neurofibrillary tangles onset in Down syndrome is relatively early and their ages are highlighted with a star (Supplementary Data 2). These graphs show that companion animals and humans possess plaques and tangles. **e** Interestingly, mature plaques are not observed in cats and other feliforme species (see names in light grey), but they are observed in dogs and their close relatives (e.g., polar bears, sea lions). These boxplots show upper and lower quartiles, maximums, and minimums. We used adult (and not juvenile) ages to assess neuropathology onset for wild Tsushima leopard cats.

Aged companion animals exhibit brain pathologies that mirror those found in humans, including plaques, tangles, vascular cerebral amyloid angiopathy (CAA), myelin disruption, lipofuscin, neuronal vacuoles, axonal degeneration, and abnormalities in cholinergic neurons^3,47,71,75,88,197^. There are discrepancies in the conclusions drawn from studies, likely due to the small sample sizes and variation in ages. We document the presence of plaques and tau pathologies in the brain and blood vessels in humans, companion animals, and wild carnivores to identify common themes across studies (Supplementary Data 2, 3^125^). Our broad survey shows that the brains of aged companion animals, as well as wild carnivores and humans, harbor Aβ plaques, tau pathology, and CAA (Supplementary Data 2^125^; Fig. 7)^45,48,75,197–202^. Despite these similarities, there are important species differences in the composition and spatial patterns of plaques and tangles (Supplementary Data 3^125^). We discuss the composition, location, and time course of plaques and tau pathology in different species.

There are similarities but also differences in plaque composition across species. Cats produce mostly small and granular Aβ deposits composed of Aβ1–42 peptides, which are similar to early-stage plaques^47,200^. In comparison, humans most often produce larger Aβ plaques composed of Aβ1–42 as well as Aβ1–40^71,88^ residues, particularly in the dense cores of mature plaques. In humans, the deposition of Aβ1–42 (but not Aβ1–40) increases with age in AD^203,204^. Dogs also have more Aβ1–42 than Aβ1–40 in their plaques, but mature plaques, with their dense Aβ1–40 cores, appear less commonly than in humans^205–211^. However, like humans, both cats and dogs have Aβ1–40 deposits in cerebral and meningeal blood vessels (Supplementary Data 2^125,200,212^) that may lead to CAA^213^.

Not all species exhibit mature plaques (Fig. 7). Dogs, but not cats, possess mature plaques. Sea lions^211,212^ and bears^206,214^, which are wild caniforme species and are more closely related to dogs than to cats, exhibit mature plaques (Fig. 7a). In contrast, wild feliforme species, including Tsushima leopard cats and cheetahs, which are more closely related to cats than to dogs, lack mature plaques^215,216^ (Fig. 7). Therefore, there may be a phylogenetic effect for the existence of plaque maturity between dogs and other caniformes versus cats and other feliformes.

Although some studies have found that Aβ deposits in the vasculature system (e.g., meningeal vessels) in advance of the neocortex in dogs^211^ and cats^217^, other studies have found the opposite order for dogs^207,209^ and cats^211^. This suggests that CAA and neocortical plaques may have different pathways^218^. Furthermore, the occurrence of CAA in feliforme species appears sparse compared with primates and dogs^200,210,216,219^. For example, there was no Aβ42 deposited in the cerebral vasculature of Tsushima leopard cats (Fig. 7). Some elderly cheetahs have Aβ42 and Aβ40 deposits in cerebral capillary vessels, but these deposits are not observed in meningeal vessels^216^. Variation in deposits within the meningeal systems could indicate species variation in the amyloid clearance system^216^.

It is unclear whether the temporal progression of plaques is similar across humans and companion animals. In humans, it is typical for AD plaques to cluster in the hippocampus before spreading to the entorhinal cortex and temporal cortex (part of the neocortex^91^), but the opposite order can occur in Down Syndrome and in non-AD neurodegenerative diseases^220,221^. Cheetahs^216^, sea lions^80^, and dogs show an early pattern of progression in the neocortices – including the frontal, temporal, and parietal cortices. Cats initially have diffuse plaques in the neocortex as well as intracellular Aβ deposits in the hippocampus^143^. Similar to humans, dogs have plaque deposits starting in a few layers of the neocortex (layers IV, V) before reaching outer layers across the cerebral cortex^8,75,88,202,207^. The spatial pattern of progression in plaques has yet to be worked out in more detail.

Plaques generally emerge at late stages of life in humans as in companion animals (Fig. 7 and Supplementary Data 2^125^). Plaques usually first occur in humans between the ages of 50–70 though they may occur earlier, as is the case in Down syndrome (Fig. 7d, e). In dog studies, half or more of the samples possess early-stage plaque pathology by age 15^206,208,210,211,222,223^. Similarly, early-stage plaques are frequently reported by age 16 in cats^71,76,88,200,217,224^ (Supplementary Data 2^125^) though cats or other feliformes do not possess mature plaques^50,59,156^ (Fig. 7e and Supplementary Data 2^125^). Despite these species’ differences, a common theme is that the onset of plaques occurs at relatively late stages of life in different species.

The presence of hyperphosphorylated tau (pTau) and NFTs has been controversial in non-human species, but a large percentage of studies report tau pathology (Fig. 7a, b). These pathologies have been found in older cats and dogs. Tau pathology can be detected by AT8 and PHF1 antibodies. There have been mixed findings when testing the AT8 antibody (Supplementary Data 2^200,225–228^). NFTs and pTau emerge by age 40–60 s in humans (Fig. 7 and Supplementary Data 2^125^). Around half of the dog and cat studies found aggregates of pTau forming NFTs in cats from 15 years or older^229^ and in dogs from as early as 13 years^207,223^ (Fig. 7 and Supplementary Data 2^125^). Tau pathology occurs in similar regions across species. In humans, tau pathology spreads across the entorhinal cortex, hippocampus, and neocortex^206,220,221^. A similar spatiotemporal pattern occurs in dogs^223,225^, cats^143,229^ and wild carnivores, including cheetahs^216^ and polar bears^230^. Collectively, our broad survey highlights that pTau and NFTs, as well as plaques, occur in cats and dogs.

## Insights from veterinary medicine for human health in old age

There are many variables to consider in selecting a model system. The study of human diseases has relied extensively on mice as model systems (Fig. 3). Rodents are easy to maintain and breed. However, many rodent studies fail to translate into meaningful treatments for humans because of the large gap that exists across the biology of mice and humans and because they are used to model human biological processes that are not clearly observed in humans. Rodents do not recapitulate many aspects of the aging process, and this is particularly pernicious in an age-related disease such as AD.

The characteristics that make mice ideal for studies (e.g., a short lifespan, large litter size, genetic manipulations) are those characteristics that sometimes make them inappropriate for expanding our understanding of human biology, physiology, and disease. Standardizing studies across human and veterinary animal work would enhance our ability to advance both fields. While experimental work on laboratory dogs is sometimes used in biomedical research and has provided important information on neurobiological mechanisms in controlled conditions^231^, our primary aim is to point to the many untapped opportunities to synergize with veterinarians who are focused on enhancing the quality of care in companion animals. Investigating how companion animals age with us in our homes could provide many insights into the field of aging. Our focus on companion animals is in line with initiatives such as the Dog Aging Project^232^.

For many years, animals have been used to detect health hazards. For example, miners in the early 20th century brought canaries into coal mines to determine whether there were toxic exposure levels to gasses (e.g., carbon monoxide, methane) that would impact the health of miners. Similarly, companion animals have been used as indicators of toxins (e.g., mercury, asbestos, lead) in our diet or in the household, also referred to as ‘animal sentinels’^204,233–236^. Companion animals are particularly useful for studying the environmental impact on human aging because they have relatively short lifespans, and they share similar environments with humans. They can therefore provide many insights into the long-term consequences of environmental hazards. Large-scale projects such as the Dog Aging Project will offer many opportunities to study how lifestyle, toxins, and medications impact the biology and cognition of aged companion animals^237–239^.

### New possibilities from the integration of veterinary and human medicine

The integration of human and veterinary medicine offers new possibilities for studying a range of biomedical problems, including aging. Companion animals consist of a pool of diverse genetic breeds, raised in a wide range of different environments, and span a wide range of habitats. This diverse pool of systems permits studying natural variation, which can be integrated with manipulations in the laboratory and in client-owned homes. For example, we can track companion animals with similar genotypes (e.g., siblings) raised in different environments (e.g., indoor versus outdoor cats) to evaluate the impact of the environment on health and disease incidence. Studying siblings raised in different environments would permit parcellating the impact of environment and the interaction between genotype and environment on health. We can also harness the broad variation across cat and dog breeds to find markers of CDS and identify conserved and species-specific risk alleles of aging. The variation and diverse range of possibilities in the laboratory and in client-owned homes stand in contrast with the limited variation within inbred mouse lines, which are constrained to be housed in the laboratory. Clinical trials in companion animals are already implemented to evaluate the impacts of drugs on companion animals, and we can integrate these findings with human medicine.

Dogs have been bred from relatively small founder populations, and they have been selected based on appearance, leading to high levels of inbreeding. Many breeds are also predisposed to inherited diseases^240–243^, but this can be less the case in some mixed breeds^244^. Evidently, the variation in allele frequency and differential predisposition to disease across dog populations makes them well-suited to uncover the genetic basis of diseases. Less recognized, however, is that dogs would be well-suited to investigate the genetic basis of individual variation in aging. The shortened lifespan of dogs relative to humans means that they are well-suited to study the interaction of genetics and environment on rates of aging, and that they can act as animal sentinels for human health.

## Insights from experiments in veterinary medicine

There are clinical trials focusing on dietary modifications and drugs aimed to improve the standard of living in aged companion animals^62^. Dietary modifications have led to improvements in animal cognition (e.g., disorientation, changes in social interactions, reduced house-soiling behavior), with improvements observed within a few weeks after the onset of treatment^245,246^. Drugs, including antidepressants (e.g., selegiline hydrochloride), are given to animals suffering from cognitive dysfunction. These drugs are effective in alleviating sleep patterns and other cognitive traits^247^. Although more work is needed to evaluate how these interventions can improve cognitive capacities integrating these experimental interventions in humans and companion animals could accelerate progress in characterizing and treating diseases of aging.

## Diverse model systems reform AD research

There is an open question as to whether AD is a disease that is an emergent property of aging or is a distinct condition that occurs during aging^16^. The field of comparative neuroscience is ideally poised to tackle these challenging possibilities. In our broad survey, we identified many commonalities in lifespan and age-related brain and behavioral changes in humans and companion animals^248^. Companion animals and humans share similar environments; they exhibit relatively extended lifespans and possess evidence of brain atrophy, plaques, and tangles in aging. Rodents, NHPs, and species from other taxonomic groups may show some of these traits, but our study highlights that companion animals and humans represent an extreme end of this spectrum in possessing a relatively extended lifespan and neuropathologies like those observed in AD.

The relative lifespan in humans and some companion animals (i.e., cats) is considerably extended relative to their wild counterparts. Several traits, including lifespan extension, similar environments, brain plaques and tangles, and other neuropathologies, are evident in companion animals and humans. These correlated traits are interesting because they may indicate the existence of underlying relationships between lifespan, brain atrophy, and neuropathologies. In this case, lifespan extension in companion animal models and humans is concomitant with brain atrophy and neuropathologies. Perhaps, lifespan extension and other traits are linked together in these distant taxonomic groups.

A broad taxonomic survey across mammals is needed to assess general links between lifespan, aging, and the emergence of AD-like phenotypes. While humans and companion animals display a constellation of similar traits such as lifespan extension and aging, there are also cases where lifespan is not necessarily concomitant with the emergence of brain atrophy and neuropathologies. Indeed, some species are long-lived but may not necessarily exhibit AD-related brain pathologies as observed in humans. For example, naked mole rats have some resistance to the toxic effects of plaques, which is unlike that in humans^249^. On the other end of the spectrum, spawning salmon, which are not known to be a long-lived species, exhibit brain pathologies that resemble those observed in AD^250^. Accordingly, aging and longevity may stimulate the likelihood of developing AD-like neuropathologies, but each species may age differently. The environment plays a role in the development of disease incidence. Shared environments across humans and companion animals, with increased medical care and subsequent survival to old age, as well as more sedentary lifestyles, may provide the conditions for the development of these neuropathologies at late stages of life.

## Conclusion

AD is sometimes considered uniquely human, in part because the hallmarks of this disease are seldom naturally occurring in rodent models and NHPs. Also, these traditional model systems tend not to survive into old age. The presence of neuropathologies in companion animals casts doubt on the notion that AD is unique to humans. Our broad survey highlights instances of convergent evolution with humans and companion animals, which have evolved a relatively extended lifespan and concomitant age-related changes in brain atrophy and neuropathologies. Therefore, companion animals are well-suited as models of human aging. The issue of identifying the most appropriate model system for a disease is one of many examples where increased integration across veterinary and human medicine could synergize to address long-standing problems in biomedical fields. A One Health perspective can enhance our comprehension of neurodegenerative diseases shared by humans and our companion animals as they age alongside us.

### Reporting summary

Further information on research design is available in the Nature Portfolio Reporting Summary linked to this article.

## Supplementary information

Supplementary Information

Description of Additional Supplementary Files

Supplementary Data 1

Supplementary Data 2

Supplementary Data 3

Reporting Summary

## Data Availability

Data in Fig. 5 are from the AnAge database (https://genomics.senescence.info/species/index.html). Data in Fig. 3 were downloaded from NCBI available (https://pubmed.ncbi.nlm.nih.gov/). Data in Fig. 7 are from Supplementary Data 2, 3, and these are available on Zenodo (10.5281/zenodo.7957575).
