# Supplementary material for: Going beyond established model systems of Alzheimer’s disease: companion animals provide novel insights into the neurobiology of aging
Source: Commun Biol. 2023 Jun 21;6:655. doi: 10.1038/s42003-023-05034-3 (PMC10284893; doi:10.1038/s42003-023-05034-3)
Supplement: Supplementary file 1 — Supplementary Information [file 42003_2023_5034_MOESM1_ESM.pdf]

Supplementary Information

Supplementary Methods

We describe the sources of the data used to assess relative usage of model systems (Figure 3) as well as the variation in maximum lifespan across species (Figure 5).

Relative usage of model systems

Data on the relative usage of different model systems are extracted from the National Library of Medicine (NCBI) (https://pubmed.ncbi.nlm.nih.gov/). We used keywords and extracted the number of articles downloaded from NCBI (Figure 3). We used these data as an index of model system usage.

Relative lifespan extension in model systems

The AnAge database includes information on the maximum recorded lifespan in different species[1]. We used the AnAge database (https://genomics.senescence.info/species/index.html) to assess whether there is evidence of extensions in maximum life span across select species (Figure 5). We compared the maximum lifespan of the domesticated species relative to other species within their genus or family. Specifically, we divided the maximum lifespan of domesticated species relative to the average maximum lifespans of other species within the same genus or family. A value greater than 1 means that the domesticated species has a relatively extended lifespan relative to other species within the same genus or family. As an example, we computed the relative maximum lifespan of humans relative to the average lifespans of great apes (n=5 species; Figure 5b) to assess whether the maximum lifespan is relatively extended in

humans relative to other great apes. The number of species for each taxonomic group are shown in Figure 5.

Neuropathologies across species

We performed a literature search to assess the prevalence of neuropathologies across humans, companion animal models, and wildlife carnivore species (i.e., sea lions, bears). We extracted demographic data including age, sample size, location, and number of individuals showing brain plaques, tangles, and cerebral angiopathology. The inclusion of wild-life carnivore species permits testing whether neuropathologies are exclusive to domesticated companion animals or whether these pathologies are general to carnivore species. We include a condensed version of our literature search in Supplementary Table 2-3.

We collated information across studies. We extracted the age and incidence of individuals with neuropathologies. These methods and samples (age, health status) used vary across studies. We specify some medical conditions, including cognitive dysfunction syndrome (CDS), Down syndrome (DS), and neurodegenerative disorders (Supplementary Table 2-3) because these clinical diagnoses should impact the age and relative number of individuals with AD-like neuropathologies. Not every study included detailed information about clinical diagnoses in their reports and that studies vary in the level of detail with which they reported demographic data. Variation in methods and samples used across studies may contribute to variation in estimated age of onset and prevalence of neuropathologies across species. It is for these reasons that we focus on large trends across studies.

Supplementary References

1. Tacutu, R. *et al.* Human ageing genomic resources: integrated databases and tools for the biology and genetics of ageing. *Nucleic Acids Res.* **41,** D1027-D1033 (2013).
