# Supplementary material for: Going beyond established model systems of Alzheimer’s disease: companion animals provide novel insights into the neurobiology of aging
Source: Commun Biol. 2023 Jun 21;6:655. doi: 10.1038/s42003-023-05034-3 (PMC10284893; doi:10.1038/s42003-023-05034-3)
Supplement: Supplementary file 2 — Description of Additional Supplementary Files [file 42003_2023_5034_MOESM2_ESM.pdf]

Description of Additional Supplementary Files

File name: Supplementary Data 1
Description: Domestication terminology and species information.

File name: Supplementary Data 2
Description: Neuropathologies and demographic information across humans, companion animals, and other carnivore species (shortened version).

File name: Supplementary Data 3
Description: Neuropathologies and demographic information across humans, companion animals, and other carnivore species (extended version).
